# Undertaking Healthy Nutrition Behaviors by Patients with Type 1 Diabetes as an Important Element of Self-Care

**DOI:** 10.3390/ijerph192013173

**Published:** 2022-10-13

**Authors:** Beata Irena Sińska, Karolina Dłużniak-Gołaska, Mariusz Jaworski, Mariusz Panczyk, Aneta Duda-Zalewska, Iwona Traczyk, Urszula Religioni, Alicja Kucharska

**Affiliations:** 1 Department of Human Nutrition, Faculty of Health Sciences, Medical University of Warsaw, 27 Erazma Ciołka Street, 01-445 Warsaw, Poland; 2 Pediatric Clinical Hospital—University Clinical Center, Medical University of Warsaw, 63A Żwirki I Wigury Street, 02-091 Warsaw, Poland; 3 Department of Education and Research in Health Sciences, Faculty of Health Sciences, Medical University of Warsaw, 14/16 Litewska Street, 00-575 Warsaw, Poland; 4 Department of Public Health, Faculty of Health Sciences, Medical University of Warsaw, Jana Nielubowicza Street 5, Blok F, 02-097 Warsaw, Poland; 5 School of Public Health, Centre of Postgraduate Medical Education of Warsaw, Kleczewska 61/63, 01-826 Warsaw, Poland

**Keywords:** healthy diet, self-control/self-care, responsibility for health, type 1 diabetes mellitus

## Abstract

Background: Self-control/self-care means the active participation of a diabetic patient in therapy. It involves making numerous decisions and undertaking actions independently. The primary activities under the patient’s control include adherence to medication regimens and maintenance of a health-promoting lifestyle, especially a healthy diet. However, the sense of responsibility for one’s own health, i.e., high sense of responsibility (HSR), is an important element in the treatment of diabetes and in undertaking pro-health behaviors. The study aimed at analyzing adherence to dietary recommendations in the context of HSR in patients with type 1 diabetes. Methods: The cross-sectional study was conducted on a group of 394 adults. The assessment of adherence to dietary recommendations was performed with the present authors’ Diabetes Dietary Guidelines Adherence Index (DDGA Index). The measurement of HSR was performed with the standardized Sense of Responsibility for Health Scale (HSRS). The assessment of the multifactorial influence of independent variables on the DDGA Index, including the “responsibility for health” variable, was conducted with the use of a linear regression model. Results: The mean DDGA value was 18.68 (SD = 3.97). The patients significantly more often avoided unhealthy products than included recommended products into the diet at a required frequency. A positive correlation was demonstrated between HSR and adherence to dietary recommendations (β_std._ = 0.43, *p* < 0.001). Conclusions: The sense of responsibility for one’s health plays a main role in adherence to dietary recommendations in diabetes. Our study showed that a higher sense of responsibility for health was associated with a higher level of adherence to dietary recommendations. Patients with a high sense of responsibility for one’s health will be more involved in the therapeutic process, including adherence to dietary recommendations. Therefore, all education actions should comprise not only dietary knowledge transfer and shaping appropriate skills, but they should also strengthen the sense of responsibility for one’s health.

## 1. Introduction

Adherence to a healthy diet is one of the more difficult steps for patients in the treatment of diabetes [1], because it includes: adherence to recommendations concerning drug administration and the maintenance of healthy lifestyle comprising regular physical activity and healthy diet [2,3,4,5,6]. According to research, numerous patients failed to adhere to the recommended diet [7,8]. In the case of type 2 diabetes, the level of non-adherence to the diet was largely variable and ranged from 2.2% to 87.5% [9,10,11].

Non-adherence to lifestyle schemes was identified as high, both in developed and developing countries, and it was associated with increased hospitalization and mortality rates [12,13,14]. Adherence to a healthy diet may be influenced by a variety of intrapersonal, interpersonal, and social factors which may be improved via the identification and removal of barriers in the patients [15].

Dietary behaviors of patients with diabetes may depend on the perceived necessity to take care of one’s health [16], which was disrupted with the development of the disease. It is believed that the above-mentioned care is directly reflected in the intensification of the sense of responsibility for one’s health (HSR) [17]. HSR should be defined as a cognitive variable referring to self-awareness and self-evaluation, concerning motivation and the use of various behavioral strategies aiming at health maintenance [18]. This psychological variable is expressed in two dimensions, i.e., active involvement and suitable behavior. The dimension of active involvement refers to cognitive and motivational aspects associated with the need to undertake suitable activities to maintain a good health status. The dimension of suitable behavior refers to actions taken to maintain or improve health. Importantly, these two dimensions complement each other [19]. Yet, HSR should be treated as a cognitive structure targeted at behaviors aiming at health maintenance. Therefore, the awareness of one’s own experiences and related consequences is particularly important in this context. Placing health in the hierarchy of one’s values is also significant [16]. 

Notably, the issue of the sense of responsibility for one’s health was analyzed in the literature, both in the context of undertaking health-promoting behaviors and the role of this variable in the treatment process. It is believed that the appropriate level of this psychological trait translates into engaging in behaviors aimed at enhancing health. Nevertheless, the mechanism of developing a high level of responsibility for one’s own health is not clear [16,17,18,19,20,21,22]. Moreover, the extent of research conducted to characterize the mechanism of action of this variable in health or disease has not been fully elucidated.

Researchers emphasized that the sense of responsibility for one’s health increased with age which might be related to the frequency of undertaking health-promoting behaviors and a higher number of chronic diseases. Moreover, HSR plays an important role in undertaking suitable nutritional behaviors [20], and the frequency of physical activity [18]. This phenomenon may be due to the reflection and higher awareness of the correlation between lifestyle and health status. A person who has a critical and reflective ability to look at the present in which he functions, allows him to take appropriate actions. Such a person is able to assess the effectiveness of actions, as well as taking appropriate activities in the future. In this context, a sense of responsibility for one’s own health can be shaped using the Gibbs’ Reflective Cycle [23]. The use of reflection in the treatment of diabetic patients is accentuated in the literature. It is emphasized that educational and reflection interventions are effective approaches for improving self-care outcomes among adults with diabetes [24,25]. It can be assumed that reflection is an important step towards shaping the sense of responsibility for their own health in patients with diabetes. However, there is a lack of such research in the literature.

Considering the above-mentioned observations it may be assumed that the sense of responsibility for one’s health will play a crucial role in the therapeutic process. It is due to the fact that a suitable level of intensity of this variable determines a higher index of motivation as regards treatment during a disease [21,26]. Preliminary research conducted on a group of patients with diabetes revealed that the sense of responsibility for one’s health played an important role in adhering to therapeutic recommendations [17,27]. However, the research was conducted on a small patient sample. Furthermore, the results mostly referred to patients with type 2 diabetes or a mixed group, i.e., including patients with diabetes type 1 and 2 [17]. The mechanism of action of the sense of responsibility for one’s health is unknown in the context of patients with type 1 diabetes. A research gap was noted in this area. Therefore, the study aimed to assess adherence to dietary recommendations in the context of the sense of responsibility for one’s health in a selected group of adult patients with type 1 diabetes.

## 2. Materials and Methods

### 2.1. Design and Participants

The observational cross-sectional online questionnaire study—Diabetes Nutritional Treatment In Adults Perception, DIANUTRA—was conducted in 2020 in a group of patients with type 1 diabetes. A non-probability sampling of study participants was used. The inclusion criteria were: age 18 and more, at least 1-year history of type 1 diabetes, and informed consent to participate in the study. The study was anonymous and participation was voluntary.

### 2.2. Data Collection

The questionnaire was distributed via the Google Forms web survey platform. The link to the questionnaire was shared via social media (Facebook) in groups of patients with type 1 diabetes (www.mojacukrzyca.org) (accessed on 1 January 2020) and the personal contacts of study group participants (the snowball method). Study supervisor initiated official talks with the administrators of social group websites which bring together patients with type 1 diabetes. The administrators were presented the assumptions of the project and consented to post links to questionnaires and send them to the members of the social group designed for patients with type 1 diabetes.

### 2.3. Ethics

The aim of the study was described in the information for patients. Prior to the study the participants were informed that it was anonymous and the data were confidential. No personal data or computer IP were collected. Due to the anonymous character of the questionnaire and no possibility to follow sensitive data, the study required no approval of the Bioethics Committee. The Bioethics Committee of the Medical University of Warsaw obtained information about the study.

### 2.4. Tools and Variables

The assessment of the adherence to dietary recommendations was performed with the present authors’ Dietary Guidelines Adherence Index (DGA Index) [28]. Diabetic patients should be encouraged to adhere to the principles of healthy nutrition developed for healthy individuals, which should additionally comprise specific needs related to the age, physical activity, complications, comorbidities, and patient preferences. The DDGA Index combines current recommendations concerning healthy nutrition for the Polish population published by the National Institute of Public Health—National Institute of Hygiene [29] and the guidelines of behavioral therapy of the Polish Diabetes Association [30]. Based on the above-described recommendations and thorough literature analysis, a group of experts in dietetics and diabetology indicated 29 groups of products and the recommended frequency of their consumption. There were 14 groups of products recommended in the diet of individuals with type 1 diabetes: (1) raw vegetables; (2) boiled vegetables and vegetable soups; (3) fresh fruits; (4) whole grain bread; (5) other whole grains: dark pasta, rice, coarse-grained groats, and natural breakfast cereals; (6) dairy products (no added sugar); (7) legumes; (8) fish; (9) unsalted nuts and seeds; (10) white meat; (11) oils; (12) margarines; (13) eggs; and (14) water. Another 15 groups included products whose consumption should be limited or replaced with healthier alternatives: (15) red and processed meat; (16) sweets; (17) salty snacks; (18) sweetened drinks; (19) fast food; (20) butter; (21) lard; (22) processed cheese; (23) sweetened dairy products; (24) refined bread; (25) other refined grains: white rice and fine-grained groats; (26) breakfast cereals; (27) flour dishes; (28) ready-made sauces and instant products; and (29) canned meat, fish, and vegetables. The respondents specified the frequency of consuming individual product groups by choosing the following answers: “several times a day”, “once a day”, “several times a week”, “1–3 times a month”, and “never”. One point was scored if the frequency of the consumption of a specific group of products adhered to the recommendations. If a response revealed no adherence to the recommendations, 0 points were scored. An additional point was scored for regular meal consumption (Table 1). DDGA Index value was expressed as the total score between 0 and 30 points. Higher DDGA Index values were interpreted as a higher degree of adherence to dietary recommendations (0 points—complete lack of adherence to the recommendations, 30 points—complete adherence to the recommendations). Based on the obtained score, the results were transformed into the standard 1–10 scale. The obtained 10-point ranges were transformed into three levels of adherence to dietary recommendations: low level (stens 1–4), moderate level (stens 5–6), and high level (stens 7–10).

The study also involved the measurement of the sense of responsibility for one’s health with a standardized questionnaire. The Sense of Responsibility for Health Scale (HSRS) was developed by Adamus [21]. The scale consists of 12 items rated on a 5-point scale (1—hardly ever, 2—rarely, 3—sometimes, 4—often, and 5—nearly always/very often). HSRS allows the determination of the total level of the sense of responsibility for one’s health (HSRS-T) and includes two subscale scores: Active Involvement (HSRS-AI) and Adequate Behavior (HSRS-AB). Only the total level of responsibility for one’s health was assessed in the present study. It is due to the fact that the HSRS-AI and HSRS-AB subscores are correlated. Cronbach’s alpha for the HSRS was 0.724.

The questionnaire also included questions concerning: the duration of the disease, type of insulin therapy (a pen/pump), insulin units used daily, insulin units per kilogram of body weight, the frequency of hypo- and hyperglycemic episodes, the knowledge of the insulin-to-carbohydrate ratio, knowledge of the number of carbohydrate exchanges consumed daily, and the calorie value of consumed diet. We also collected sociodemographic data including: age, gender, place of residence, and the level of education. Moreover, based on the height and current body weight declared by the respondents we calculated body mass index (BMI) which was interpreted in accordance with the WHO classification [31]. 

### 2.5. Data Analysis

Quantitative and categorical variables were described with the methods of descriptive statistics. The following measures were determined for quantitative variables: central tendency (mean, M) and dispersion (standard deviation, SD). The following measures were determined for categorical variables: number (*n*) and frequency (%).

Continuous variables were converted into categorical variables by transforming the scores obtained with the DDGA Index into the standard 10-point scale. The obtained ranges of 10 stens were then used to determine three levels of the assessed variable: low level (stens 1–4), medium level (stens 5–6), and high level (stens 7–10). The comparisons of the determined three groups as regards the occurrence of various variants of categorical variables being the potential factors associated with the DDGA Index were performed with the non-parametric chi-squared test. The comparison of qualitative variables was performed with one-way analysis of variance (ANOVA) with the Fisher’s Least Significant Difference post hoc test.

The assessment of the multifactorial influence of independent variables on the DDGA Index, including the “responsibility for health” variable was performed with the use of a linear regression model. The estimation of model parameters was calculated with the method of least squares. We determined standardized regression coefficients (β_std._) with 95% confidence intervals (CI) in order to estimate the directions and strength of correlations between variables. Adjusted R^2^ value was determined to assess the degree of variance by the assessed regression model.

All calculations were performed with STATISTICA TM 13.3 software (TIBCO Software, Palo Alto, CA, USA). For all analyses, the *p*-level of <0.05 was considered statistically significant.

## 3. Results

### 3.1. Patient Characteristics

The study was conducted in a group of 394 adults with a minimum 1-year history of type 1 diabetes. The average age of study participants was 35.34 years (SD = 11.36). Women (*n* = 256, 65.0%), individuals with tertiary education (*n* = 225, 57.3%), and the inhabitants of cities (*n* = 181, 45.9%) constituted the marked majority of the respondents. Selected demographic variables and those associated with the course and treatment of the disease in the study group were presented in Table 2.

### 3.2. Adherence to Dietary Recommendations

The analysis of adherence to individual dietary recommendations in the behavioral therapy of type 1 diabetes revealed that the considerable majority of patients adhered to recommendations concerning the avoidance of unhealthy products (the mean percentage of adherence to recommendations concerning “unhealthy products” reached 80.4%). Adherence to the desired dietary behaviors by the study group was considerably poorer, as the mean percentage of adherence to the recommendations concerning healthy products was only 44.1%. Detailed presentation of adherence to dietary recommendations is included in Table 3.

**Figure 1:**
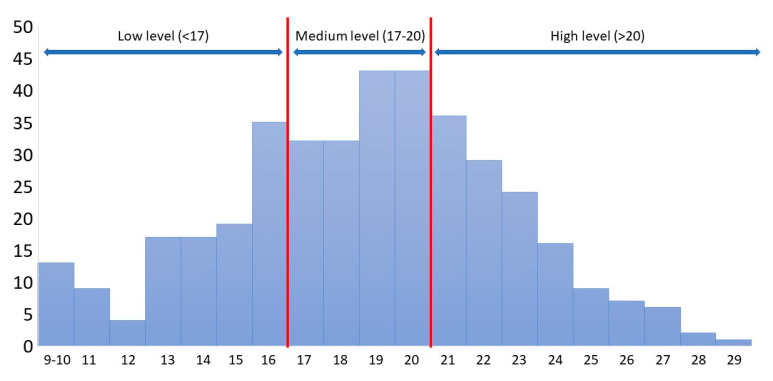
The results of the measurement obtained for the adherence by diabetic patients to the dietary recommendations of the DDGA Index (determined score ranges based on the transformation to the standard 10-point scale). Red line: the range of medium level.

The mean value of the DDGA Index was 18.68 (SD = 3.97) in the study group with the minimum of 9 points and maximum of 29 points. A low level of adherence to recommendations (stens 1–4) was obtained by 28.9% of the respondents (*n* = 114), a medium level (stens 5–6) by 37.8% of the respondents (*n* = 149), and a high level (stens 7–10) by 33.3% of the respondents (*n* = 131). The characteristics of the DDGA Index scores obtained in the study group were presented in Figure 1.

### 3.3. Factors Influencing Adherence to Dietary Recommendations

**Figure 2:**
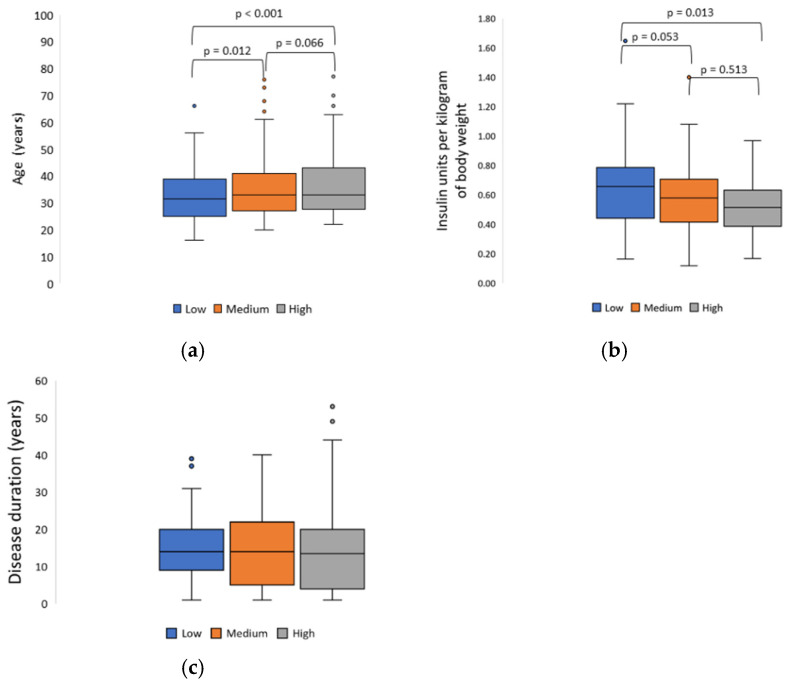
(**a**) Mean age (F = 8.755, *p* < 0.001), (**b**) mean insulin dose per kilogram of body weight (F = 3.364, *p* = 0.036), (**c**) mean disease duration (F = 0.004, *p* = 0.996) depending on the level of adherence to dietary recommendations (the *p*-values for the Fisher’s Least Significant Difference post hoc test are reported).

The analysis of correlation between selected demographic factors and those associated with the course of the disease and adherence to dietary recommendations revealed that a higher adherence was characteristic of patients with a tertiary education (*p* < 0.001), those with fewer hypoglycemia episodes during the week (*p* = 0.028), those who knew the average number of consumed CEs during the day (*p* = 0.001) and individuals who knew the calorie value of their daily diets (*p* < 0.001) (Table 4). Better adherence to recommendations was also noted in older patients (*p* < 0.001) and those who used fewer insulin units per kilogram of body weight (*p* = 0.036) (Figure 2).

### 3.4. Responsibility for Health

The results of the sense of responsibility for one’s health indicated that the average level of this variable in the study group patients was 47.43 (SD = 6.94) with a minimum of 20.0 points and a maximum of 64.0 points and the maximum total being 65.0. Moderately left-sided skewed distribution of the variable was noted (skew = −0.33), which means a slight advantage of high results over low ones. No outlier data were found in the obtained results.

### 3.5. Responsibility for Health and Adherence to Dietary Recommendations

**Figure 3:**
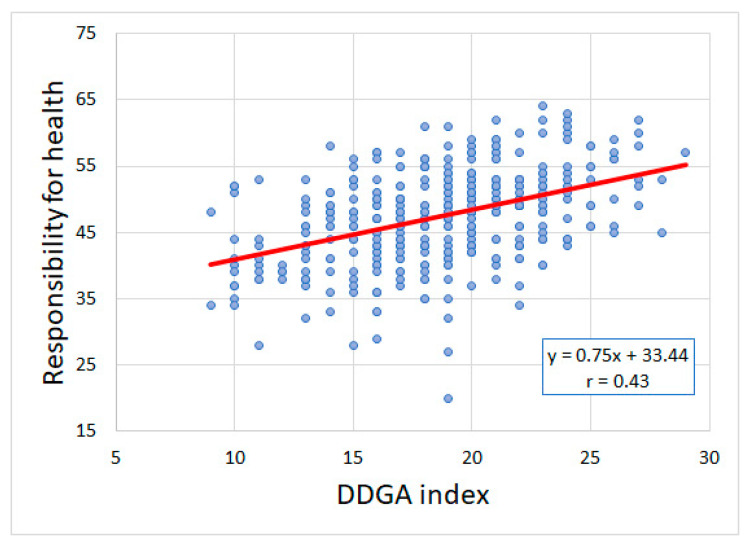
Responsibility for health and adherence to dietary recommendations (r-Pearson = 0.43, *p* < 0.001). Red line: it is a trend.

The assessment of the correlation between responsibility for health and adherence to dietary recommendations revealed a positive correlation between those two variables (β_std._ = 0.43, *p* < 0.001, Figure 3). Adjusted β_std._ was 0.30 (*p* < 0.001) after comprising additional covariates in the regression model (Table 5). A higher sense of responsibility for health was associated with a higher level of adherence to dietary recommendations.

Covariates which, apart from responsibility for health, had a positive influence on the level of adherence to dietary recommendations were: age (β_std._ = 0.10, *p* = 0.037), female sex (β_std._ = 0.16, *p* = 0.001), the knowledge of the average quantity of consumed CEs (β_std._ = 0.10, *p* = 0.026), the knowledge of the calorie value of everyday diet (β_std._ = 0.17, *p* < 0.001), and tertiary education (β_std._ = 0.09, *p* = 0.049). A regression model comprising covariates explained the total of 29% of dependent variables (adherence to dietary recommendations) with the standard error of estimate of 3.37.

## 4. Discussion

The study revealed that the psychological variable, i.e., the sense of responsibility for one’s health, played an important role in adhering to dietary recommendations by patients with type 1 diabetes. The observation is consistent with general reports concerning the role of the sense of responsibility for one’s health in undertaking health-promoting behaviors in the general population [17], and in individuals with chronic disease [21].

According to the obtained results, the sense of responsibility for one’s health is a property which enhances positive involvement into the therapeutic process. It is associated with the fact that the sense of responsibility has a positive influence on health-related decisions and behaviors of individuals. Three important aspects should be considered in this context. They include: role responsibility, which means the care of one’s body and satisfying one’s biological needs; causal responsibility, which manifests as choosing behaviors that have a beneficial effect on health status and disease prevention; responsibility based on liability, e.g., costs and undesirable effects of diseases or injuries [16]. Therefore, it may be assumed that the appropriate degree of the sense of responsibility for one’s health supports shaping a positive attitude towards treatment and adhering to dietary recommendations. It translates into undertaking adequate actions as regards treatment and increases the effectiveness of introduced therapy [32]. This hypothesis was confirmed by the present results. The results showed that a higher sense of responsibility for health was associated with a higher level of adherence to dietary recommendations.

Notably, the sense of responsibility for one’s health is not a constant variable. The discussed psychological variable undergoes modifications. It starts to be shaped during adolescence and is related to the concept of psychosocial development by Erik Erikson. The discussed theory refers to human development throughout the life seen as the sequence of stages when some kinds of crisis situations typical of certain ages are solved or remain unsolved. Erikson [33] emphasized that the period of adolescence determined the formation of normal relationships with others via shaping one’s identity and, therefore, might be translated into the need to care not only for one’s own health, but also for the health of other individuals important for a specific person (e.g., family members). In this context, caring for one’s health means practicing a healthy lifestyle on a daily basis, eliminating behaviors that are risky for health and participation in creating conditions promoting health maintenance [16].

Shaping a sense of responsibility for one’s own health requires psychological effort and confronting reality. This confrontation does not always bring a pleasant change. In this context, self-awareness plays a key role. A person must answer the question about the goal of his own actions, and define his own expectations. Skills reflection is important in this process. In this context, Gibbs’ Reflective Cycle can be used [23]. The model has six stages, usually displayed as follows: Description, Feelings, Evaluation, Analysis, Conclusion, and Action Plan. These six steps make up the cycle. 

Initially, the patient has to describe his current diet. However, this description must be prepared by the patient. Not by a dietitian. A dietitian may act as a support in this process, but may not be the main actor. The patient himself has to assess his current diet and adherence to dietary recommendations.

The analysis of the degree of adherence to dietary recommendations by the study group of patients with type 1 diabetes revealed a markedly higher percentage of individuals (80.4%) declaring the avoidance of unhealthy products compared to those who adhered to healthy dietary behaviors (44.1%).

The mean DDGA Index value obtained by the study group of patients was 18.68 points (64.0%), with the maximum of 29 points (100%). As a comparison, a cross-sectional study conducted by Katsaridis et al. in a group of 162 patients with type 2 diabetes revealed a low level (41.2%) of adherence to the dietary recommendations of the American Diabetes Association (ADA) [34]. Moreover, three subgroups were distinguished in the whole group of patients: those adhering to the recommendations at a low (28.9%), medium (37.8%), and high (33.3%) level. Notably, the subgroup of patients in which adherence to recommendations was insufficient was the smallest.

In the next step, the patient defines his emotions related to being on a diet. This is a crucial stage because long-term adherence to dietary recommendations is difficult to achieve which was also demonstrated by other researchers [35,36,37].

This process takes time and commitment. It should be noted that reflection gives patients an opportunity to express their experiences and personal difficulties with diabetes, as well as to enable them to participate actively in their care process [38]. It allows people to become aware of their emotions and express them through words [39]. Subsequently, the person can take actions to deal with these emotions. For example, emotions associated with a chronic disease, such as diabetes [24]. For this reason, a dietary message should be as positive as possible and indicate the wide variety of options of individualized composition of the diet within the scope of a diabetic diet. Negative information should be limited and it should only refer to situations in which the necessary restrictions/elimination of specific food products was well demonstrated in clinical trials [19].

Strengthening the sense of responsibility for one’s own health through reflection should take into account the patient’s age. For example, adolescence is also a period during which type 1 diabetes is most commonly diagnosed. A young person has to deal with not only developmental crises, but also with crises related to the diagnosis of a chronic disease, i.e., type 1 diabetes. Therefore, it seems justified to provide adequate support to patients with type 1 diabetes and to implement an appropriate psychological approach during nutrition education. Such an approach will let patients shape a positive attitude towards treatment process and adhere to therapeutic recommendations. Therefore, the obtained results indicate the necessity of the suitable preparation of educators dealing with nutritional counseling for patients with type 1 diabetes. Counseling should involve strengthening the sense of responsibility for one’s health via reflecting on and paying attention to the expectations and needs of the patient. Thus, the appropriate selection of educational content and its adaptation to the current needs of the patient seems justified with the simultaneous strengthening of the need to care for one’s health. The process of nutrition education should be based not only on providing basic information on disease treatment, but it should also strengthen the personality dispositions of the patient.

The analysis of the role of psychological variables in the nutrition therapy of patients with type 1 diabetes is the strength of the study. Moreover, it is the first study which focused on the role of the sense of responsibility for one’s health. Previous research was conducted in a group of patients with type 2 diabetes or in mixed groups—type 1 and 2 diabetes. The present authors also developed the Diabetes Dietary Guidelines Adherence Index (DDGA Index) as a part of the study, which facilitated the rapid assessment of adherence to dietary recommendations in patients with type 1 diabetes. The index comprises specific dietary recommendations for people with diabetes, so it may help assess how well a patient adheres to the recommended dietary pattern. It needs to be emphasized that the present study focused on a group of adult patients who suffered from type 1 diabetes. A considerable majority of research on type 1 diabetes was focused on children and adolescents.

The present study is not devoid of limitations. Cross-sectional studies do not allow the complete determination of the cause-and-effect relationship. They also do not enable the assessment of the influence of psychological variables on dietary behaviors over time. Another limitation is related to the procedure of patient selection for the study. Differences in the gender structure and the wide age range of the participants constitute significant limitations. Another important limitation is the lack of data related to the patients’ lifestyle or monthly income of the family. Therefore, further research is needed to verify the relationship between various factors and the quality of diet of the patients with type 1 diabetes. It needs to be highlighted that the patients declared the frequency of consuming particular groups of products.

## 5. Conclusions

Adherence to dietary recommendations in type 1 diabetes is an enormous challenge. Due to its chronic character, the disease is associated with numerous difficulties and barriers which must be constantly overcome by the patient. The predictors of adherence to dietary recommendations are an important area of research, as their identification may contribute to the higher effectiveness of behavioral therapy. The sense of responsibility for one’s health is an important condition of dietary behaviors. The present study showed that a higher sense of responsibility for health was associated with a higher level of adherence to dietary recommendations. Therefore, it is justified to develop and implement educational actions for patients with type 1 diabetes. Such actions should comprise not only knowledge transfer and shaping of appropriate skills, but also strengthen the sense of responsibility for one’s health. Patients with a high sense of responsibility for one’s health will be more involved in the therapeutic process, including adherence to dietary recommendations.

## Figures and Tables

**Table 1:** The components of Diabetes Dietary Guidelines Adherence Index (DDGA Index).

Group of Products	Recommended Consumption Frequency
Healthy groups of products	
(1) Fresh vegetables	several times a day
(2) Boiled vegetables and vegetable soups	once a day or more
(3) Fresh fruits	once a day or more
(4) Whole grain bread	once a day or more
(5) Other whole grains	once a day or more
(6) Dairy products (no added sugar)	once a day or more
(7) Legumes	several times a week or more
(8) Fish	once a week or more
(9) Unsalted nuts and seeds	several times a week or more
(10) White meat	several times a week or less
(11) Oils	once a day or more
(12) Margarines	once a day or more
(13) Eggs	several times a week or more
(14) Water	several times a day
Unhealthy groups of products	
(15) Red and processed meat	once a week or less
(16) Sweets	once a week or less
(17) Salty snacks	once a week or less
(18) Sweetened beverages	once a week or less
(19) Fast food	1–3 times a month or less
(20) Butter	once a week or less
(21) Lard	once a week or less
(22) Processed cheese	several times a week or less
(23) Sweetened dairy products	once a week or less
(24) White bread	once a day or less
(25) White rice, fine-grained groats	once a day or less
(26) Refined grains/Breakfast cereals	once a week or less
(27) Flour dishes	once a week or less
(28) Ready-made sauces and/or instant products	1–3 times a month or less
(29) Canned meat/fish/vegetables	once a week or less
Healthy nutritional habits	
(30) Consume meals at regular times	all of them/some of them

**Table 2:** Characteristics of the study group (*n* = 394).

Gender, *n* (%)	
female	256 (65.0)
male	138 (35.0)
Age (years)	
M ± SD	35.34 ± 11.36
Min-Max	18.0–77.0
Education, *n* (%)	
Primary/vocational	23 (5.9)
Secondary	145 (36.9)
Tertiary	225 (57.3)
Place of residence, *n* (%)	
Countryside	88 (22.3)
Small town	125 (31.7)
Big city	181 (45.9)
Disease duration (years)	
M ± SD	14.88 ± 10.71
Min-Max	1.0–54.0
BMI categories, *n* (%)	
<18.5 kg/m^2^	5 (1.3)
18.6–24.9 kg/m^2^	208 (52.8)
25.0–29.9 kg/m^2^	126 (32.0)
30.0–34.9 kg/m^2^	40 (10.2)
>35.0 kg/m^2^	15 (3.8)
Type of insulin therapy, *n* (%)	
Insulin pen	221 (56.1)
Insulin pump	173 (43.9)
Insulin units per day	
M ± SD	43.04 ± 19.32
Min–Max	10.0–140.0
Insulin units per kilogram of body weight	
M ± SD	0.58 ± 0.22
Min–Max	0.1–1.6
Hypoglycemia episodes, *n* (%)	
every day	23 (5.8)
5–6 times a week	35 (8.9)
4–3 times a week	78 (19.8)
1–2 times a week	123 (31.2)
only once	70 (17.8)
never	65 (16.5)
Hyperglycemia episodes, *n* (%)	
every day	74 (18.8)
5–6 times a week	75 (19.0)
4–3 times a week	87 (22.1)
1–2 times a week	72 (18.3)
only once	47 (11.9)
never	39 (9.9)
Knowledge of insulin/CEs ratio, *n* (%)	320 (81.2)
Knowledge of CEs consumed daily, *n* (%)	261 (66.2)
Knowing the calorie value of one’s diet, *n* (%)	170 (43.1)

M—mean, SD—standard deviation, CEs—carbohydrate exchanges.

**Table 3:** Adherence to dietary recommendations (*n* = 394).

Group of Products	Percentage of Respondents Who Adhered to Recommendations (%)
Healthy groups of products	
(1) Fresh vegetables	27.4
(2) Boiled vegetables and vegetable soups	25.6
(3) Fresh fruits	54.3
(4) Whole grain bread	49.0
(5) Other whole grains	22.8
(6) Dairy products (no added sugar)	26.1
(7) Legumes	18.0
(8) Fish	56.9
(9) Unsalted nuts and seeds	37.3
(10) White meat	87.6
(11) Oils	23.1
(12) Margarines	16.8
(13) Eggs	57.6
(14) Water	81.5
Mean percentage of respondents who adhered to healthy product recommendations	44.1
Unhealthy groups of products	
(15) Red and processed meat	80.2
(16) Sweets	53.8
(17) Salty snacks	81.2
(18) Sweetened beverages	83.5
(19) Fast food	75.9
(20) Butter	50.8
(21) Lard	98.0
(22) Processed cheese	79.4
(23) Sweetened dairy products	73.6
(24) White bread	83.5
(25) White rice, fine-grained groats	99.5
(26) Refined grains/Breakfast cereals	88.6
(27) Flour dishes	74.9
(28) Ready-made sauces and/or instant products	91.4
(29) Canned meat/fish/vegetables	91.9
Mean percentage of respondents who adhered to unhealthy product recommendations	80.4
Healthy nutritional habits	
(30) Consume meals at regular times	77.9

**Table 4:** Factors influencing adherence to dietary recommendations (*n* = 394).

Variable	Low Level		Medium Level		High Level		χ^2^	*p*-Value *
	*n*	%	*n*	%	*n*	%		
Gender								
Woman	72	63.2	99	66.4	85	64.9	0.307	0.858
Man	42	36.8	50	33.6	46	35.1		
Education								
Primary/vocational	11	9.6	8	5.4	4	3.1	24.427	0.000
Secondary	58	50.9	52	34.9	35	26.9		
Tertiary	45	39.5	89	59.7	91	70.0		
Place of residence								
Village	35	30.7	27	18.1	26	19.8	6.678	0.154
Town	32	28.1	51	34.2	42	32.1		
City	47	41.2	71	47.7	63	48.1		
BMI categories (kg/m^2^)								
<18.5	1	0.9	1	0.7	3	2.3	6.398	0.603
18.6–24.9	58	50.9	75	50.3	75	57.3		
25.0–29.9	35	30.7	51	34.2	40	30.5		
30.0–34.9	14	12.3	15	10.1	11	8.4		
>35.0	6	5.3	7	4.7	2	1.5		
Type of insulin therapy								
Pens	62	54.4	91	61.1	68	51.9	2.567	0.277
Insulin pumps	52	45.6	58	38.9	63	48.1		
Hypoglycemia episodes								
Every day	5	4.4	12	8.1	6	4.6	6.182	0.800
5–6 times a week	8	7.0	16	10.7	11	8.4		
4–3 times a week	21	18.4	30	20.1	27	20.6		
1–2 times a week	38	33.3	48	32.2	37	28.2		
Only once	23	20.2	21	14.1	26	19.8		
Never	19	16.7	22	14.8	24	18.3		
Hyperglycemia episodes								
Every day	28	24.6	28	18.8	18	13.7	20.121	0.028
5–6 times a week	28	24.6	24	16.1	23	17.6		
4–3 times a week	15	13.2	31	20.8	41	31.3		
1–2 times a week	19	16.7	35	23.5	18	13.7		
Only once	14	12.3	17	11.4	16	12.2		
Never	10	8.8	14	9.4	15	11.5		
Knowledge of insulin/CEs ratio								
No	27	23.7	25	16.8	22	16.8	2.527	0.283
Yes	87	76.3	124	83.2	109	83.2		
Knowledge of average CEs consumed daily, *N* (%)								
No	54	47.4	42	28.2	37	28.2	13.292	0.001
Yes	60	52.6	107	71.8	94	71.8		
Knowing the calorie value of one’s diet								
No	85	74.6	82	55.0	57	43.5	24.280	0.000
Yes	29	25.4	67	45.0	74	56.5		

CE—carbohydrate exchange; * chi-squared test.

**Table 5:** Regression model for the influence of selected factors on adherence to dietary recommendations (F(11, 375) = 15.132, *p* < 0.001).

Independent Variable	b	β_std._	−95% CI	+95% CI	t	*p*-Value
Intercept	10.32				4.847	0.000
Responsibility for health	0.17	0.30	0.21	0.40	6.317	0.000
Age (years)	0.03	0.10	0.01	0.19	2.098	0.037
Gender						
Male (ref.)						
Female	0.69	0.16	0.06	0.27	3.205	0.001
BMI	−0.03	−0.03	−0.17	0.11	−0.440	0.660
Disease duration	−0.02	−0.10	−0.21	0.00	−1.931	0.054
Insulin units per day	−0.02	−0.07	−0.37	0.23	−0.480	0.631
Insulin units/kg/bw	−0.81	−0.04	−0.32	0.23	−0.322	0.748
Knows the average CE consumption	0.43	0.10	0.01	0.19	2.234	0.026
Knows the calorie value of the diet	0.68	0.17	0.08	0.26	3.613	0.000
Education						
Primary/vocational (ref.)						
Secondary	−0.31	−0.04	−0.13	0.05	−0.968	0.334
Tertiary	0.61	0.09	0.00	0.19	1.977	0.049

ref.—reference level, b—non-standardized regression coefficient, β_std._—standardized regression coefficient, CI—confidence interval for β_std_.

## Data Availability

All data is available from the corresponding author.
